# The Medical Professionalism of Korean Physicians: Present and Future

**DOI:** 10.1186/s12910-015-0051-7

**Published:** 2015-08-26

**Authors:** Soojung Kim, Sookhee Choi

**Affiliations:** Department of Medical Humanities and Social Sciences, School of Medicine, The Catholic University of Korea, 222 Banpodaero, Seocho gu, Seoul 137-701 ROK

## Abstract

**Background:**

Medical professionalism is a core aspect of medical education and practice worldwide. Medical professionalism must be reinterpreted to adapt to different social/cultural/historical contexts. We conducted a survey to examine the current understanding and perceived value of medical professionalism among Korean physicians.

**Methods:**

The survey was distributed to 950 physicians nationwide; 721 (75.89 %) completed surveys were returned between 1 April and 31 July 2011.

**Results:**

In their practice, Korean physicians prioritized the values and virtues of medical professionalism in the following (descending) order: veracity, respect for patient autonomy, integrity, responsibility, altruism, and honesty. Approximately two-thirds of physicians responded that medical professionalism is an element of their vocation. When asked to choose the most important sets of attributes or virtues of medical professionalism from a provided list, the top three sets (in descending order of frequency) were: “responsibility and duty,” “veracity, integrity, and honesty,” and “rapport with patients and conversational skill.”

**Conclusions:**

Korean physicians value moral duties, such as responsibility and veracity, more than they do moral virtues, such as altruism and honesty with patients. It is presumed that physicians are under pressure due to governmental regulation of the national healthcare system and have difficulty exercising their autonomy.

**Electronic supplementary material:**

The online version of this article (doi:10.1186/s12910-015-0051-7) contains supplementary material, which is available to authorized users.

## Background

Medical professionalism is a representative term that embodies the nature, obligations, and identity of medical doctors [1]. The importance of medical professionalism has been emphasized in medical education [2–7] because of the misconduct and improper ethical attitudes reported in recent newspaper articles in Korea [8–11]. The medical profession has been perverted to careerism that seems to guarantee good income and secure job status, instead of a professional vocation with defined virtues that is a vocation [12].

We start with the question of Korean physicians’ understanding of medical professionalism. Traditional medicine had been practiced for many centuries in Korea before Western medicine was introduced. Asian medical practitioners have traditionally been expected to be concerned healers who cure sicknesses of body and soul; indeed, they are also respected for these virtues.

Doctors in general are recognized as professionals who carry out a socially important role, similar to those of clergy and lawyers [13]. An understanding of medical professionalism has been assumed to be transferred informally from mentor physicians to young physicians without an official education [14]. However, current medical education is oriented towards medical skills, competency, and evidence-based science, and the importance of medical professionalism has been neglected in Korean medical education because of a lack of an appropriate text and teaching methodology [15–19].

Western medicine was introduced to Korea during a short period of modernization at the time of Japanese colonization [17, 18]. It emphasizes the physician’s scientific knowledge and medical skill; that is, competence is valued over moral attitude. In this approach, the physician–patient relationship is not considered an important element in acquisition of the medical good: the patient’s healing. The compassion of the physician for the patient’s vulnerability is taken for granted in Confucian culture. However, this approach has been criticized as paternalistic as medical consumerism in Korea has favored a focus on the patient’s rights. Moreover, industrialization encourages the pursuit of personal profit, and an altruistic attitude and social responsibility have been disregarded as outdated Confucian values [19].

According to an old saying, the good doctor treats diseases of the body; the better doctor heals sickness of the soul; and the great doctor cures both of these and societal illness as well. The individual and collective obligations of physicians are to restore the health of the individual and of society [20]. The essence of physicians lies in their role as both healers and professionals [14]. The primary role of a physician is to care for sick people and restore their health. Physicians have a social responsibility: they must be concerned about public health issues and understand the societal expectations of the medical profession. However, the notion of social responsibility of physicians as medical professionals has, until recently, rarely been discussed in Korea.

Public concern about medical professionalism and medical ethics has increased due to two dramatic events in Korean medical history [21]. The first was a legal case involving a patient’s spouse who secured the patient’s discharge from hospital against the physician’s advice, after which the patient died. The patient’s brother sued the hospital, the physician for medical negligence. This event reveals the conflict between social expectations of physicians and physicians’ own views of appropriate behavior. The public regarded the case as irresponsible behavior by the physician that caused the premature death of the patient. In contrast, physicians viewed the case as a violation of the physician’s autonomy. The second case was a physicians’ strike against a governmental regulation that stipulated separation of the privileges of prescribing and dispensing drugs. Physicians understood this move as impairment to medical professionalism, whereas the public felt it was a safeguard against abuse of power by the medical profession. The need for increased medical professionalism has been voiced by both the public and the medical profession for the last two decades, and the medical societies have called for medical professionalism to be taught and restored as a feature of the profession [22].

The roles and responsibilities of physicians must be redefined, and medical ethics must be promoted among Korean physicians. Physicians must understand these roles and responsibilities towards patients, their peers, and society if medical professionalism is to be reestablished in Korean society. The purpose of this study was to demonstrate, through physicians’ ratings of importance of the various values or virtues of medical professionalism, that even physicians who are not familiar with the term ‘medical professionalism’ embody the concept through their history of devotion to medical practices that are consistent with these values.

## Method

This study was based on an anonymous survey (see Additional file 1) of Korean physicians about their understanding of medical professionalism as a feature of their vocation. Medical professionalism encompasses all of the essential qualities that doctors must possess. However, the survey was designed to investigate only the overall attitudes and morals of Korean doctors, because there is no question that all doctors are required to have medical knowledge and the clinical ability to practice medicine. We focused on the six crucial virtues of medical professionalism addressed in numerous studies—patient autonomy, responsibility, honesty, altruism, veracity, and integrity. The protocol and survey were approved by the IRB of Songeui Medical Campus, the Catholic University of Korea in March 2011(Approval Number CUMC11U043). The Cronbach’s alpha for a pilot study of 37 physicians in Seoul was 0.87. The importance of each answered item was rated on a 5-point scale, and mean responses for each item were compared. The survey was distributed nationwide to 950 physicians, and 721 (75.89 %) returned completed surveys between April 1 and July 31, 2011. The survey was distributed to physicians in university hospitals, local hospitals, and clinics, the names and addresses of which were acquired from web postings, telephone directories, and colleagues.

The survey started with questions about familiarity with “medical professionalism” and about medical professionalism as a vocational feature of medicine. The various virtues of medical professionalism were not described explicitly, but were expressed within descriptions of different clinical contexts. Since each physician might understand each virtue differently, specific situation in which each virtue is implied is presented instead of each particular virtue. Each item was intended to imply one of the following aspects of medical professionalism: veracity, patient autonomy, integrity, responsibility, altruism, and honesty [1, 23–25]. The survey also asked respondents to choose a set of core values of medical professionalism and to offer methods for promoting medical professionalism among Korean physicians.

## Results

Table 1 presents the findings regarding physicians’ general conceptualizations of medical professionalism. Most physicians (67 %, 486/721) responded that medical professionalism is a feature of the vocation of medicine (see Table 1). There were age-related differences (*p* < .0001) in this finding, with the highest frequency of affirmative responses about professionalism among those in their 60s and the lowest ratings among those in their 20s (see Table 2). Comparison of particular items between those who advocated medical professionalism as a vocational feature and those who did not is also important, but is beyond the scope of this study.

**Table 1:** The percetion of medical professionalishm as a vocation (unit: person/%)

Factor	Strongly Yes		Yes	Mean	Not at all likely		Never
Medical professionalism is a vocation	200 (27.74)		286 (39.67)	130 (18.03)	98 (13.59)		7(0.97)
		486 (67.41)				235 (32.59)	
Religious belief affects medical professionalism	131 (18.17)		323 (44.80)	154 (23.36)	100 (13.87)		13 (1.80)
		454 (62.97)				267 (37.03)	
Medical professionalism has some connection with belief in vocation	112 (15.53)		348 (48.27)	170 (23.58)	83 (11.51)		8 (1.11)
		460 (63.80)				261 (36.20)

**Table 2:** The level of perception of medical professionalism and vocation among age groups in Korean physicians (unit: person/%)

Factor	Age					*P*-value
	20–29	30–39	40–49	50–59	60–69	
	(*N* = 43)	(*N* = 217)	(*N* = 183)	(*N* = 213)	(*N* = 65)	
I have heard of medicao professionalism before	16 (37.21)	79 (36.41)	78 (42.62)	123 (57.75)	38 (58.46)	<.0001
Medical professionalism is a vocation	18 (41.86)	130 (59.91)	131 (71.58)	156 (73.24)	51 (78.46)	<.0001
Religious belief affects medical professionalism	22 (51.16)	111 (51.15)	122 (66.67)	150 (70.42)	49 (75.38)	<.0001
Medical professionalism has some connection with belief in vocation	20 (46.51)	116 (53.46)	119 (65.03)	153 (71.83)	52 (80.00)	<.0001

As Table 3 shows, Korean physicians prioritized the values of medical professionalism in their practice in the following order: veracity, respect for patient autonomy, integrity, responsibility, altruism, and honesty.

**Table 3:** Core virtues in medical professionalism in Korean physicians (unit: person/%)

Variables	Min	Max	Average	S. D.
Veracity	2.00	5.00	4.29	0.71
Integrity	1.50	5.00	3.44	0.63
Altruism	1.33	5.00	3.09	0.57
Responsibility	1.00	5.00	3.43	0.76
Honesty	1.00	4.25	2.77	0.49
Autonomy	2.00	5.00	3.79	0.60
Total	2.20	4.40	3.31	0.31

Veracity and honesty are thought to have similar meanings. But, veracity in this context refers to the particular matter of truth-telling to patients or patients’ families regarding medical errors of self or other colleagues; hospital infections; and the diagnosis, development, and prognosis of disease. Veracity requires a relationship of mutual respect and reliance between patients and physicians [26]. ‘Honesty’ was used here to refer to medical practice without any deception. In Korea, the national health insurance service determines and authorizes the price and cost of each particular order or treatment in medical practice, and physicians are aware of the orders and treatments that are reimbursed by the service. Medical decisions that deviate from the normally reimbursable orders or treatments can result in referral to the health insurance review and assessment service, which will often levy an enormous fine against the physician and request that the bill be returned to the patient. Korean physicians tend to complain that national health insurance coverage restricts their professional discretion and encourages their unethical behaviors. In particular, the health insurance service’s preference for reducing the number of claims can discourage physicians from recommending and implementing the most appropriate treatments. Some physicians recommend unnecessary and expensive tests to patients for financial reasons, and some physicians make slight changes in diagnoses so that patients can receive health insurance coverage [20].

Those who responded that medical professionalism is a feature of the medical vocation rated the six values in the following (descending) order of importance: veracity, respect for patient autonomy, responsibility, integrity, altruism, and honesty (see Table 4).

**Table 4:** Core virtues in medical professionalism in Korean physicians who answered medical professionalism is a vocation (unit: person/%)

Variables	Min.	Max.	Average	S. D.
Veracity	2.00	5.00	4.37	0.67
Integrity	1.50	5.00	3.49	0.64
Altruism	1.33	5.00	3.14	0.58
Responsibility	1.50	5.00	3.53	0.76
Honesty	1.25	4.00	2.79	0.49
Autonomy	2.00	5.00	3.84	0.58
Total	2.40	4.40	3.37	0.30

The physicians were asked to choose the most important component sets for medical professionalism: “responsibility and duty” (549 respondents, 76.46 %), “veracity, integrity, and honesty” (411 respondents, 57.24 %), “rapport with patients and conversational skill” (264 respondents, 36.77 %), “benevolence, altruism, and compassion” (252 respondents, 35.10 %), “trustworthiness” (192 respondents, 26.74 %), “public service” (163 respondents, 70 %), “patient autonomy” 9,120 respondents, 16.71 %), “virtue and good heart” (108 respondents, 15.04 %), and “collegial relationships” (95 respondents, 13.23 %) (see Table 5).

**Table 5:** Frequencies of the important professionalism virtues for physician (unit:person/%)

Question	Frequency	Percentage (%)
Honesty, integrity, veracity	411	57.24
Responsibility, duty	549	76.46
Benevolence, altruism, compassion	252	35.10
Relation iwth colleagues	95	13.23
Service for the public	163	22.70
Respect for patient’s autonomy	120	16.71
Rapport with patient, narration	264	36.77
Trusthwothiness	192	26.74
Virtuous mind, good heart	108	15.04

The respondents presented the following suggestions for promoting medical professionalism and boosting values among Korean physicians: a continuing professional development (CPD) incentive and CPD credits; reinforcement of medical ethics activation of regional medical societies; reorganization of medical insurance and adequacy of medical insurance reimbursements; use of role models; emphasis on medical professionalism and self-regulation within medical associations; reinforcement of doctors’ involvement in and relationship with professional societies; public service; and cooperation between Western and Asian medicine (see Table 6).

**Table 6:** Methods to improve medical professionalism

Answer	Frequency	Percentage (%)
Proposal of role models	42	11.05
Incentiv of CPD & CPD credits	192	50.53
Service for the public	24	6.32
Activating regional medical society	53	13.95
Reorganization of medical insurance & adequacy of insured medical fee	50	13.16
Emphasis on medical professionalism & self regulation	41	10.79
Cooperation of western & eastern medicine	10	2.63
Reinforcement of medical ethics	131	34.47
Reinforcement of doctor’s involvement & relation with society	36	9.47

## Discussion

The term “medical professionalism” implies elements of both “medicine” and “professionalism.” Medicine is delivered by doctors who have professed to use their medical knowledge and skill only for the good of the patient. Medicine as a profession is maintained by doctors who develop, comply with, and embody the medical code [27]. Therefore, medical professionalism is maintained and enriched by the virtuous person who makes a lifelong commitment to maintain integrity as a doctor. Starr stated that “A profession is an occupation [that] regulates itself through systematic, required training and collegial discipline; that has a base in technical specialized knowledge; and has a service rather than a profit orientation, and is enshrined in its code of ethics” [28]. Indeed, medical professionalism is a complex concept that defies a unitary definition. Medical professionalism is classified not as an singular domain but as a multidisciplinary or interdisciplinary domain that includes medical ethics and medical humanities as well as medical science [6, 23].

Cruess et al. [29] emphasized the relationship between a profession and a vocation. According to Cruess, a profession is a vocation (calling) that requires specialist knowledge and skills acquired through training and education, and the professional is expected to use these characteristics to serve others. To be involved in a profession means to promise to follow ethical codes such as expertise, integrity, morality, altruism, and promotion of the common good. These duties are the basis of a contract between society and the profession. The society grants exclusive rights to the professional and the privilege of self-governance to those who adhere to the profession’s code of conduct. Therefore, a professional society and its members have accountability to the particular community they serve, to society as a whole, and to the profession. Cruess expanded the definition of a professional and applied it to medical professionalism. He argued that a doctor who has acquired, trained, and served with professional knowledge and skills and who complies with the medical ethics code is ensured the status and protection that society affords the medical profession. Therefore, the definition of medical professionalism has been developed not only by medical professionals but also with the input of society. Defining medical professionalism strictly within medical societies may cause limitations and bias. Because even major figures in a medical society may be unfamiliar with the wider social context of professionalism, they might overlook important traits or exhibit bias when defining medical professionalism [5]. Society empowers medical professionals by granting them appropriate privileges and responsibilities. Major medical society figures represent competency, appropriate roles, and responsibility for both patients and society as essential qualities that the medical profession needs to convey to society.

Wynia et al. [30] identified the characteristics of medical professionalism by analyzing three elements of the relationship between doctors and society. First, professionalism defines necessary moral duty as a commitment to medical service and societal values. Second, commitment to medical service and values results in the professionals following a code of ethics, which eventually contributes to establishment of an ethical professional culture. Third, these attitudes help medical professionals maintain their professionalism by emphasizing the importance of professional ethics in the public domain. The ethical culture of the medical profession functions as a moral ground on which medical professionals defend healthcare values and exercise political leadership to resolve various social conflicts related to medicine.

The Royal Academy of Medicine has stated that a strong motivation to be devoted to others is the primary characteristic required for a medical professional [25]. According to the Academy’s understanding of medicine as a vocation, a good doctor “is an interdisciplinary hybrid person who has the moral duty and passion to treat patients with the necessary scientific and medical skills.” The sociologist Freidson [31] also holds that professional labor could be a secular calling or vocation and not a religious one. Therefore, a good doctor who embodies a high level of medical professionalism is one who practices medicine as a vocation, regardless of personal acknowledgement for doing so.

According to the survey, over two-thirds of the physicians responded that medical professionalism is properly a part of the profession of medicine. Factors highly correlated with this response were physician experience and age. As Aristotle defined virtue as a stable disposition that is formed through habitual acts, physician’s professionalism is formed throughout medical practice over the years. Pelligrino noted that a virtuous physician is not simply born, but is formed through years of deliberate practice. Time and experience are essential factors to nurture, cultivate, and promote medical virtues. As physicians accumulate practice, they are more likely to regard medical professionalism as an element of their vocation [32].

Moral duty and responsibility were the component set that was advocated most highly as indicative of medical professionalism. Part of the survey included cases involving medical moral dilemmas in practice to determine the virtues most valued by Korean physicians. Regardless of whether the respondents endorsed medical professionalism as an element of the vocation, they listed veracity, respect for patient autonomy, integrity, responsibility, altruism, and honesty as important characteristics of medical professionalism. Korean physicians tended to emphasize moral norms, such as veracity and respect for patient autonomy, rather than altruism, as basic virtues or ordering principles of medical professionalism. The survey results reflect the current status of medical professionals in a country with a national health insurance system and a government-led healthcare policy. Korean physicians are overwhelmed with excess documents and are expected to comply with ever-changing policies. Therefore, physicians find it difficult to exercise their discretion and display the virtues of a medical professional; they feel a lack of autonomy as professionals despite their altruistic mindset.

The Royal College of Physicians has stated that all doctors must abandon their sense of supremacy, autonomy, and privilege as medical professionals [25]. However, apprehensions about status and autonomy among Korean medical professionals are somewhat different from those among doctors in the UK. The national health insurance system and strict implementation of medical law restrain medical practice and result in Korean physicians’ struggling to maintain a sense of professional integrity. When physicians feel restrained and unable to exercise their autonomy, they become defensive and remain passive in medical practice. Such behavior tarnishes the value of the medical profession, and could have detrimental effects on patient care. Furthermore, health and medical services may deteriorate as a result. In particular, both patients and doctors become victims in the event of a medical accident [33, 34]. Doctors may find it difficult not only to exercise autonomy, but also to establish a code of ethics and contribute to social justice.

It is presumed that the life-long experience of doctors gives them insight and wisdom, which is consistent with Aristotle’s virtue ethics. According to Aristotle, time and experience are essential to acquire appropriate virtue in practice [35]. It is expected that experienced physicians tend to be good at communicating with patients, which helps them acquire an integral perspective of medicine. It is also consistent with Ho’s (2011) research on the virtues of medical professionalism in non-Western countries [36]. Ho questioned whether medical professionalism developed by Western physicians could be adapted to non-Western countries with different social, historical, and cultural backgrounds, and found a difference between the core virtues of medical professionalism in Western countries and those in Chinese culture. Confucian thought is popular among people influenced by Chinese culture, and Ho concluded that its core value, Zi Zhong (conducting oneself with dignity), is similar to integrity in medical professionalism. This also explains why Korean physicians in their 50s and 60s, who one would expect to be more influenced by Confucian culture, placed a higher priority on integrity than did other age groups.

In our study, Korean physicians reported responsibility and obligation as core medical professionalism virtues in their practice, which reflects essential virtues according to the medical code of ethics. However, the physicians also recognized the importance of altruism and empathy as traditional medical professionalism virtues and reported that they realize these virtues in practice whenever the sociocultural conditions facilitate it. In conclusion, physicians tended to have an appropriate attitude toward medical professionalism, yet they felt that the social environment did not enable achievement of their potential as medical professionals. The finding that Korean physicians hold the more normative values like responsibility and veracity in higher esteem than other virtues reflects their lack of autonomy due to excess governmental limitations in the healthcare system. The health insurance review and assessment service evaluates the ‘adequacy’ of treatment provided to the patient [37]. The ‘adequacy’ evaluation identifies whether the treatment errs on the side of too much or too little, according to the tendency of most hospitals’ treatment and referral decisions for similar cases. Korean physicians are forced to comply with health insurance review and assessment service referrals rather than finding the best available and appropriate treatment for a particular patient. Assurance of physicians’ discretion is essential for patient satisfaction and quality of care [38].

The Korean healthcare system is experiencing a dilemma between extending health care insurance coverage and effectively managing the healthcare insurance budget. The government forces physicians to reduce healthcare service spending rather than raising the level of reimbursement. The degree and quality of healthcare service for a particular patient should left primarily to the physician’s medical expertise and his or her conscience. Government’s reinforcement of regulations and monitoring of physicians’ decisions is not the best way to ensure ethical behaviors in medical practice. To promote ethical culture, it is necessary to allow physician’s autonomy and to encourage a self-policing system within the medical profession [38]. According to medical law, article 26, the Korean Ministry of Health and Welfare has the right of administrative sanctions and license management. But the Ministry only passively recognizes the self-determination of the Medical Association and exercises disciplinary action by judicial authorities due to practical reasons such as labor shortages [39]. The Medical Association’s initiative and active participation is required to reform moral culture in medical practice.

The survey results demonstrate the necessity of implementing a CPD program to reinforce the autonomy of the medical profession and to promote medical professionalism in Korea. Physicians’ autonomy requires responsible behaviors by physicians as moral agents. Therefore, the Medical Association should provide physicians with opportunities for continuing education and training to promote ethical behavior. Dissemination of ethical culture among physicians will change societal attitudes toward medical professionals, build trusting and respectful relationships, and contribute to a sound medical culture.

The survey results showed that physicians do not recognize the importance of values such as “collegial relationships” or “public service,” and these findings reflect a lack of collective medical professionalism, i.e., an inability and unwillingness to exercise self-governance, to nurture professional attitudes among colleagues, and to establish a moral culture as a professional group. Transformation based on social change is inevitable for Korean physicians and medical societies. As medicine cannot exist apart from society, the social responsibilities of physicians must be emphasized more than has been the case previously.

The survey also showed that Korean physicians are aware of the need to strengthen the ethical elements of medical professionalism and proposed CPD as a means of achieving this. Implementing a CPD program for medical professionalism, including vocation, competence, ethical elements, and leadership, will enable doctors to maintain a moral basis for their work. Such a program could lead to establishment of a new and improved medical professionalism in Korea. It is time to initiate a movement for new medical professionalism in Korea. In this way, patient trust and the reliance of society on the medical community can be restored [25]. It is encouraging that physicians who responded to the survey agreed on the necessity of CPD to promote medical professionalism. An effective CPD program will assist physicians to recognize the relationship between medical professionalism and the vocation and will help produce a sound medical culture. The physicians suggested reinforcing medical ethics as an additional way of promoting medical professionalism, which reflects their recognition of the medical ethics crisis. Social blame for the unprofessional behavior of some physicians has caused public concern over the apparent lack of medical professionalism in present-day Korea. Therefore, a CPD program based on the relationship between medical professionalism and the medical vocation should consider both medical ethics and bioethics.

A solid education program is important to produce good doctors who practice medicine in an exemplary manner. In particular, the program should emphasize the teaching of students and the medical professionalism of young physicians. Nevertheless, just as making a good doctor is similar to making a good person, designing such a program to do either is challenging [40]. The Catholic University of Korea operates an outstanding program known as the “Omnibus Program,” which has the goal of “producing good doctors” [41]. The program began in 2007 and is an empathic and holistically oriented humanities curriculum. A good doctor from a humanities perspective includes not only knowledge of scientific medicine but also of being a “humane doctor.” It is important to teach medical ethics and humanities as the foundation for developing medical professionalism; therefore, a continuing CPD program for practicing physicians should also include studies in the humanities.

## Conclusion

Korea has a relatively short history of medical professionalism, which is not yet entrenched in medical education or in the clinical context. The medical community must recognize the importance of appropriate identification and implementation of medical professionalism for Korea in the 21st century. Contemporary medical professionalism should address social concerns including public healthcare and social welfare, as well as the healing relationship between patient and physician. The country has a national health insurance system and a government-oriented health policy. Korean physicians need more opportunities to exercise self-governance and autonomy to be socially responsible for medical education and medical practice. A medical profession equipped with autonomy and independence will contribute to formation of a good medical culture.

Medical professionalism is the basis for trust in the patient–doctor relationship, and is a core element of being a good doctor [40–42]. The traditional virtues of medical professionalism, such as altruism, honesty, integrity, and competency, must be maintained. Professionalism in medicine involves an ongoing conversation about what it means to be a good doctor [43]. Patients need good doctors—doctors who make patient care their primary concern, who are competent, keep their knowledge and skills up to date, establish and maintain good relationships with patients and colleagues, are honest and trustworthy, and act with integrity and within the law [44].

As mentioned in the Health Foundation’s new definition of medical professionalism, medical practice involves not only doctors but also all other healthcare professionals. doctors and patients has become more of a partnership [45, 46, 47, 48]—one that is essential for creating patient-centered medicine [49]. However, our study targeted only physicians and did not investigate the public’s understanding of medical practice in Korea. As medicine is practiced within a particular social and cultural context, further studies should explore the perspectives of other stakeholders, such as patients, their families, governmental officials, and other medical professionals. Different opinions and appropriate feedback from various parties will contribute to establishment of a new and solid medical professionalism in Korea.

## Additional file

Additional file 1:
**Choi’s Questionnaire.** (DOCX 14 kb)
