# Supplementary material for: The Medical Professionalism of Korean Physicians: Present and Future
Source: BMC Med Ethics. 2015 Aug 26;16:56. doi: 10.1186/s12910-015-0051-7 (PMC4550064; doi:10.1186/s12910-015-0051-7)
Supplement: Additional file 1: — Choi’s Questionnaire. (DOCX 14 kb) [file 12910_2015_51_MOESM1_ESM.docx]

Choi’s Questionnaire

Medical Professionalism as a Component of the Medical Vocation of Physicians in Korea

1. Have you heard of the term "medical professionalism"?

Yes No

2. Medical professionalism is a component of the medical vocation.

1. Strongly Agree 2. Agree 3. Maybe 4. Disagree 5.Strongly Disagree

3. A doctor’s religious persuasion will have an effect on her or his medical professionalism.

1. Strongly Agree 2. Agree 3. Maybe 4. Disagree 5.Strongly Disagree

4. There is a correlation between "medical professionalism" and the individual’s professional sense of mission.

1. Strongly Agree 2. Agree 3. Maybe 4. Disagree 5.Strongly Disagree

5. I advise patients or their guardians about the doctors’ potential liability regarding nosocomial infection.

1. Strongly Agree 2. Agree 3. Maybe 4. Disagree 5.Strongly Disagree

6. I advise patients about the occurrence of medical malpractice even if such malpractice will not harm the patient’s health .

1. Strongly Agree 2. Agree 3. Maybe 4. Disagree 5.Strongly Disagree

7. I report malpractice by fellow professionals upon discovery.

1. Strongly Agree 2. Agree 3. Maybe 4. Disagree 5.Strongly Disagree

8. I allow terminal cancer patients to select their treatment method.

1. Strongly Agree 2. Agree 3. Maybe 4. Disagree 5.Strongly Disagree

9. Little gifts or entertainment offered by pharmaceutical companies will affect physicians’ practice.

1. Strongly Agree 2. Agree 3. Maybe 4. Disagree 5.Strongly Disagree

10. I treat a patient who needs care even if I have an important family function to attend.

1. Strongly Agree 2. Agree 3. Maybe 4. Disagree 5.Strongly Disagree

11. I offer sufficient explanation about risks and side effects before invasive surgery.

1. Strongly Agree 2. Agree 3. Maybe 4. Disagree 5.Strongly Disagree

12. When I treat patients, I am concerned about their ability to pay their medical expenses.

1. Strongly Agree 2. Agree 3. Maybe 4. Disagree 5.Strongly Disagree

13. If one of my long-time patients with AIDS asks me to perform a surgery on him or her, I will do so.

1. Strongly Agree 2. Agree 3. Maybe 4. Disagree 5.Strongly Disagree

14. I respect a patient's decision, even if it differs from my opinion and judgement as a medical professional.

1. Strongly Agree 2. Agree 3. Maybe 4. Disagree 5.Strongly Disagree

15. In Korea, fair medical services and benefits are being offered regardless of a patient's ability to pay.

1. Strongly Agree 2. Agree 3. Maybe 4. Disagree 5.Strongly Disagree

16. I respect the patient's autonomy while providing care.

1. Strongly Agree 2. Agree 3. Maybe 4. Disagree 5.Strongly Disagree

17. I purposely offer unnecessary check-ups to improve the financial condition of the clinic.

1. Strongly Agree 2. Agree 3. Maybe 4. Disagree 5.Strongly Disagree

18. I record diagnoses slightly differently on insurance claims if it will benefit the patient.

1. Strongly Agree 2. Agree 3. Maybe 4. Disagree 5.Strongly Disagree

19. I would accept a request to serve a place classified as a war zone, a disaster relief areas, or a SARS / Bird Flu area.

1. Strongly Agree 2. Agree 3. Maybe 4. Disagree 5.Strongly Disagree

20. I choose the following 3 as the most important requirements of medical professionals besides medical knowledge and skill.

______________

1. honesty, integrity, veracity 2. responsibility, duty

3. benevolence, altruism, compassion 4. relationship with colleagues

5. service for the public 6. patients autonomy

7. rapport with patients, narration 8. trustworthiness

9. virtuous mind, good heart

21. Please list a few suggestions for continued medical education to increase its value in promoting medical professionalism.

(Examples: role models, credit or certification in an ethics education program, revitalization of local medical associations, etc.)

____________________________________________

Thank you
